# Strong Phylogeographic Structure in a Sedentary Seabird, the Stewart Island Shag (*Leucocarbo chalconotus*)

**DOI:** 10.1371/journal.pone.0090769

**Published:** 2014-03-10

**Authors:** Nicolas J. Rawlence, Charlotte E. Till, R. Paul Scofield, Alan J. D. Tennyson, Catherine J. Collins, Chris Lalas, Graeme Loh, Elizabeth Matisoo-Smith, Jonathan M. Waters, Hamish G. Spencer, Martyn Kennedy

**Affiliations:** 1 Allan Wilson Centre for Molecular Ecology and Evolution, Department of Zoology, University of Otago, Dunedin, New Zealand; 2 Laboratory of Molecular Anthropology, School of Human Evolution and Social Change, Arizona State University, Tempe, United States of America; 3 Canterbury Museum, Christchurch, New Zealand; 4 Museum of New Zealand Te Papa Tongarewa, Wellington, New Zealand; 5 Department of Marine Science, University of Otago, Dunedin, New Zealand; 6 Department of Conservation, Dunedin, New Zealand; 7 Allan Wilson Centre for Molecular Ecology and Evolution, Department of Anatomy, University of Otago, Dunedin, New Zealand; University of Arkansas, United States of America

## Abstract

New Zealand's endemic Stewart Island Shag (*Leucocarbo chalconotus*) comprises two regional groups (Otago and Foveaux Strait) that show consistent differentiation in relative frequencies of pied versus dark-bronze morphotypes, the extent of facial carunculation, body size and breeding time. We used modern and ancient DNA (mitochondrial DNA control region one), and morphometric approaches to investigate the phylogeography and taxonomy of *L. chalconotus* and its closely related sister species, the endemic Chatham Island Shag (*L. onslowi*). Our analysis shows *Leucocarbo* shags in southern New Zealand comprise two well-supported clades, each containing both pied and dark-bronze morphs. However, the combined monophyly of these populations is not supported, with the *L. chalconotus* Otago lineage sister to *L. onslowi*. Morphometric analysis indicates that *Leucocarbo* shags from Otago are larger on average than those from Foveaux Strait. Principal co-ordinate analysis of morphometric data showed substantial morphological differentiation between the Otago and Foveaux Strait clades, and *L. onslowi*. The phylogeographic partitioning detected within *L. chalconotus* is marked, and such strong structure is rare for phalacrocoracid species. Our phylogenetic results, together with consistent differences in relative proportions of plumage morphs and facial carunculation, and concordant differentiation in body size and breeding time, suggest several alternative evolutionary hypotheses that require further investigation to determine the level of taxonomic distinctiveness that best represents the *L. chalconotus* Otago and Foveaux Strait clades.

## Introduction

The blue-eyed shags (*Leucocarbo* spp.) are a species-rich seabird clade exhibiting a circumpolar Southern-Hemisphere distribution. Numerous locally endemic taxa are associated with particular islands or island groups [1]. Within the New Zealand region there are six species currently recognised: New Zealand King Shag (*L. carunculatus*), Stewart Island Shag (*L. chalconotus*), Chatham Island Shag (*L. onslowi*), Auckland Island Shag (*L. colensoi*), Campbell Island Shag (*L. campbelli*), and Bounty Island Shag (*L. ranfurlyi*) [2].

**Figure 1:**
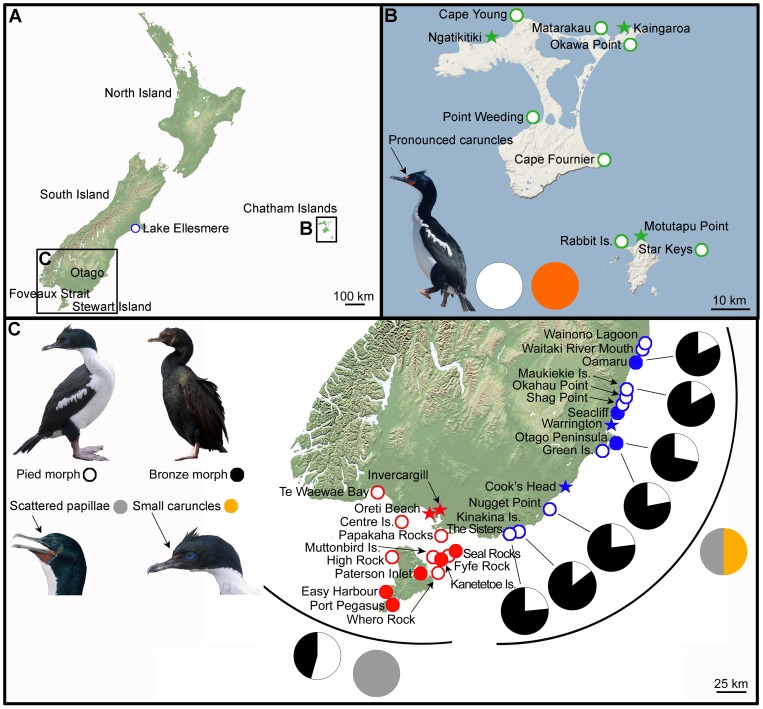
Distributional and morphological data for Chatham Island (*Leucocarbo onslowi*) and Stewart Island (*L. chalconotus*) shags. **A**: Map of New Zealand showing the location of the Chatham Islands and Otago/Foveaux Strait study sites; **B**: Distribution of *L. onslowi* (green) breeding colonies and roosting sites. Unfilled circles represent existing but un-sampled colonies and roosting sites. Filled green stars represent Holocene fossil bones at sites without colonies or roosts nearby. *Leucocarbo onslowi* exhibits pied plumage only (white pie graph) with pronounced bright orange caruncles in breeding plumage (orange pie graph). **C**: Distribution of *L. chalconotus* from Otago (blue) and Foveaux Strait (red) breeding colonies and roosting sites. Filled circles represent samples from colonies and roosting sites, while the stars represent beach wrecks found at sites without colonies or roosts nearby. Unfilled circles represent existing but un-sampled colonies and roosting sites. Shags from the Otago populations have 20–30% pied morphs (pie graphs; black: dark-bronze; white: pied) and 50%:50% small bright orange caruncles: dark to dull orange scattered papillae in breeding plumage (pie graph; yellow: small bright orange caruncles; grey: dark to dull orange scattered papillae) compared to 50–60% pied and dark to dull orange scattered papillae in breeding plumage in shags from the Foveaux Strait populations [4]–[5], [13]. In **C**, multiple breeding colonies and roosting sites are represented at the following locations (from north to south): **Warrington** and Karitane; **Otago Peninsula** including Long Beach, Aramoana, Otago Harbour, Taiaroa Head, Boulder Beach, Papanui Beach, Allans Beach, Wharekakahu Island and Gull Rocks; **Seal Rocks** and Ruapuke Island; **Easy Harbour** and Shag Rock.

**Figure 2:**
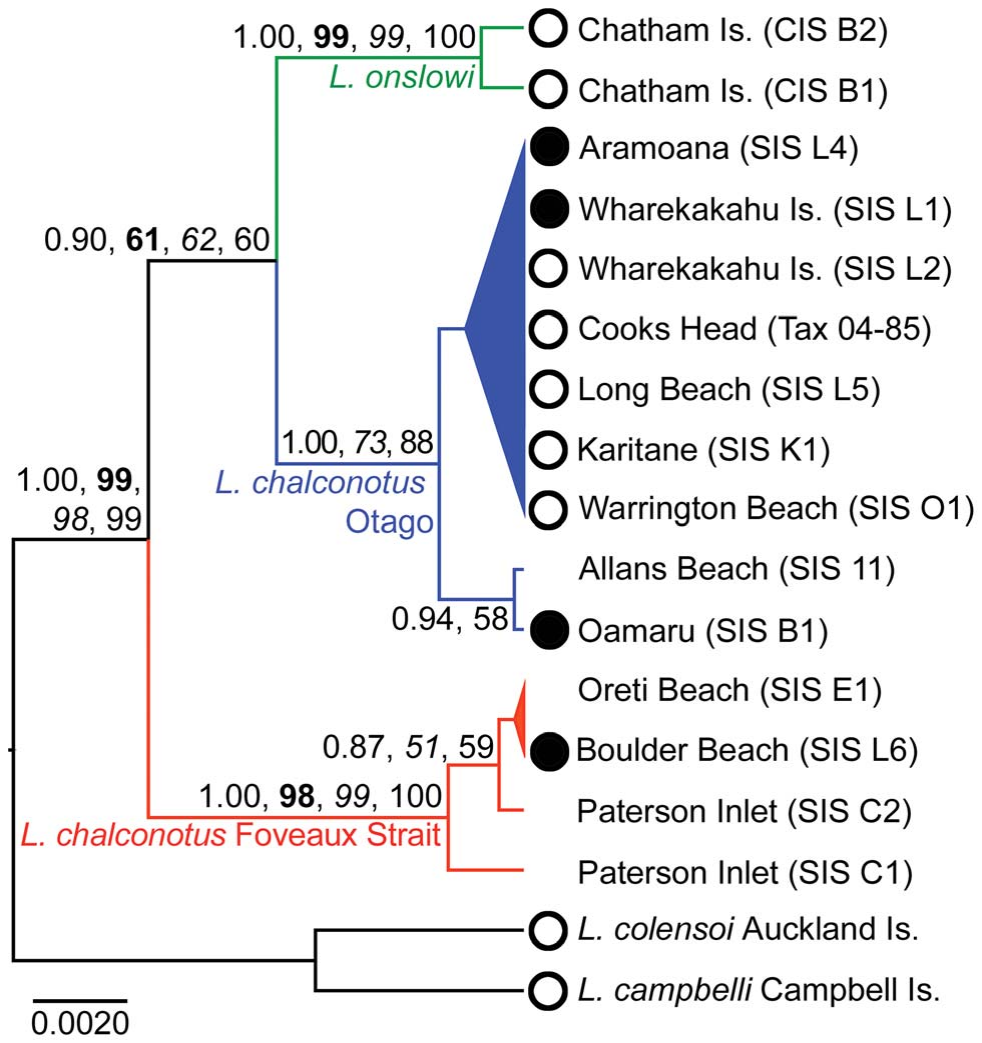
Phylogeny of *Leucocarbo chalconotus* and *L. onslowi* shags based on 1040 bp mtDNA *CR1*. Branch lengths are proportional to the number of substitutions. The maximum clade credibility tree was generated in BEAST v1.7.4 using the HKY + I model of nucleotide substitution. The phylogeny was rooted using the Auckland Island Shag (*L. colensoi*) and Campbell Island Shag (*L. campbelli*). For clarity, only posterior probability (PP) and bootstrap support (**bold**: maximum likelihood; *italics*: maximum parsimony; Roman: neighbour joining) values for major clades are shown (>0.60 PP). Nodes with less than 0.60 PP have been collapsed. Dark-bronze (black circle) or pied (white circle) plumage morphotype, if known, has been indicated on the phylogeny.

**Figure 3:**
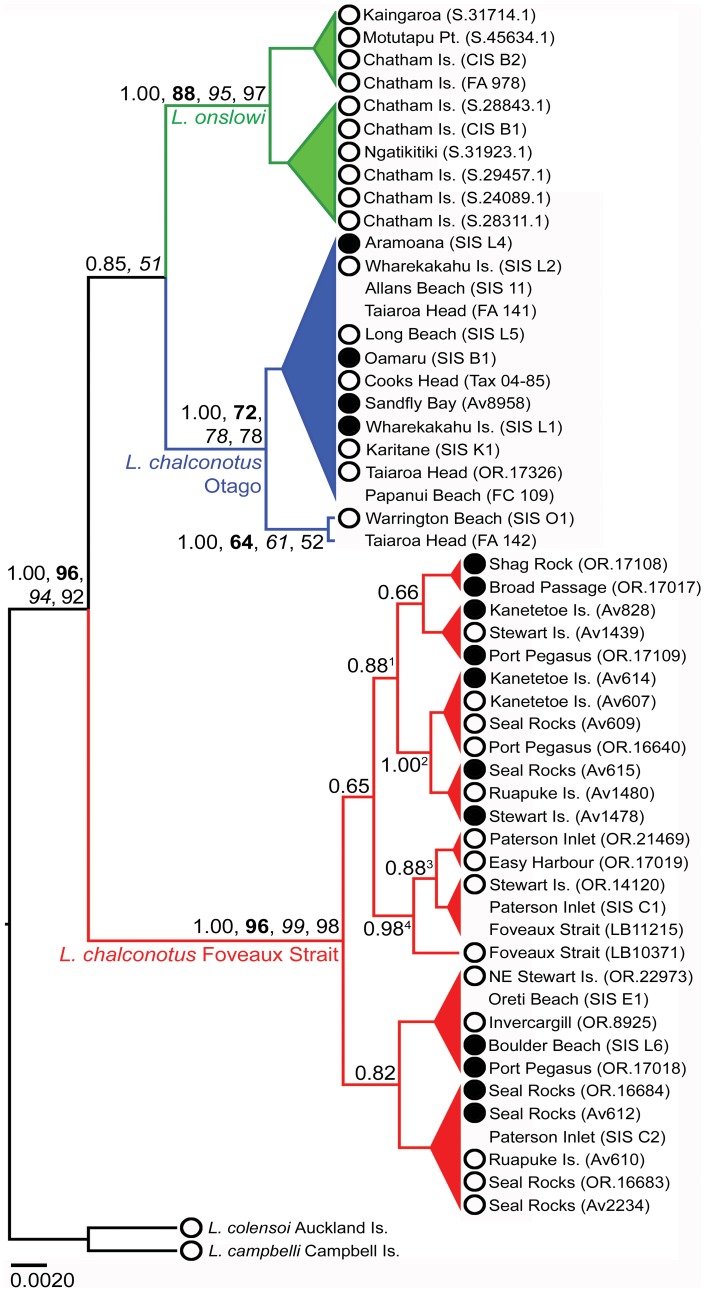
Phylogeny of *Leucocarbo chalconotus* and *L. onslowi* shags based on 248 bp mtDNA *CR1*. Branch lengths are proportional to the number of substitutions. The maximum clade credibility tree was generated in BEAST v1.7.4 using the HKY + I model of nucleotide substitution. The phylogeny was rooted using the Auckland Island Shag (*L. colensoi*) and Campbell Island Shag (*L. campbelli*). For clarity, only posterior probability (PP) and bootstrap support (**bold**: maximum likelihood; *italics*: maximum parsimony; Roman: neighbour joining) values for major clades are shown (>0.60 PP). Nodes with less than 0.60 PP have been collapsed. Weakly supported alternative branching patterns are not shown. Dark-bronze (black circle) or pied (white circle) plumage morphotype, if known, has been indicated on the phylogeny. ^1^: 0.88, **55**, *53*; ^2^: 1.00, **75**, *78*, 82; ^3^: 0.88, **62**, *55*, 65; ^4^: 0.98, **68**, *59*, 73.

The Stewart Island Shag *L. chalconotus* is endemic to southern New Zealand, with its current range extending from the Stewart Island region (Foveaux Strait) north to the Otago coast (Fig. 1). This species is unique within its genus in that it shows substantial intraspecific variation in plumage colouration, with distinct pied and dark-bronze morphotypes (Fig. 1). Both colour morphs are widespread throughout the species' range, with no evidence for assortative mating associated with plumage phenotype [3]. Nevertheless, morphological and behavioural analyses by Lalas [4], and Lalas and Perriman [5], have detected consistent regional differentiation between Otago and Foveaux Strait populations of *L. chalconotus* (Fig. 1), primarily associated with relative frequencies of pied versus dark-bronze morphotypes, the extent of facial carunculation, body size and breeding time. They found that, (1) the Otago group comprises 20–30% pied morphs versus 50–60% in the Foveaux Strait group (Fig. 1); (2) Otago birds have approximately equal frequencies of small facial caruncles versus scattered papillae, whereas Foveaux Strait birds always have scattered papillae (Fig. 1); (3) morphometric analyses of museum specimens revealed birds from Otago were 7–14% larger than those from Foveaux Strait; and (4) breeding was initiated sooner in Otago (May-September versus September onwards in Foveaux Strait) [4]–[5].

The current Otago breeding populations of *L. chalconotus* range from Maukiekie Island south to Kinakina Island, whereas the southern breeding populations are restricted to islands in Foveaux Strait (Fig. 1). The current non-breeding range for the Otago group extends from Lake Wainono south to The Sisters in the Catlins (Fig. 1) [5], with rare vagrants observed as far north as Lake Ellesmere [6]. Historically, the Otago populations were centered near Otago Peninsula [7]–[9]. During the 1980s, Otago breeding colonies were located at Maukiekie Island and several sites around Otago Peninsula with a roost site at Nugget Point [4] (Fig. 1). Recent decades, however, have seen substantial northward (e.g. roost site at Oamaru) and southward (e.g. breeding site at Kinakina Island, roost site at the The Sisters in the Catlins) expansions of this regional population [5]–[6] (Fig. 1). This expansion results from an overall population increase and is apparently driven by *L. chalconotus*' ability to readily abandon and establish new breeding colonies, and large numbers at some breeding colonies creating overflow into adjacent regions [5].

There is no obvious contemporary physical barrier between the two regional groupings of *L. chalconotus* populations, and it has been suggested that the species may have been more widespread in the past. Indeed, bones attributable to *Leucocarbo* have been found in Holocene fossil sand dune deposits and early Maori archaeological sites (ca. 1280–1450 AD) throughout the current range of *L. chalconotus* and further north along the eastern South Island, suggesting a former widespread distribution prior to Polynesian settlement of New Zealand ca. 1280 AD [10]–[12].

The taxonomic history of *L. chalconotus* has been complicated because many of its external morphological character states are shared with other blue-eyed shag taxa. In breeding plumage the combination of caruncle size and colour is diagnostic with *L. carunculatus* having a large yellow caruncle and *L. onslowi* having a pronounced bright orange caruncle [4], [13]. In *L. chalconotus* from Otago the small caruncles are usually bright orange, whereas when there are only scattered papillae, these are dark to dull orange. Likewise, the scattered papillae in *L. chalconotus* from Foveaux Strait are dark to dull orange [4], [13]. Caruncle size decreases and its colour fades in non-breeding plumage, so there is currently no reliable method for discriminating between *L. carunculatus*, *L. onslowi*, and the pied morphs of *L. chalconotus* in the wild [14]. *L. chalconotus* and *L. onslowi* have previously been regarded as either subspecies of *L. carunculatus* [4], [15]–[16] or as full species [1]–[2], [17]–[19]. Siegel-Causey [18] proposed osteological character state changes that united his New Zealand blue-eyed shags, but was unable to distinguish between *L. chalconotus* and *L. onslowi*, due to small sample size and a lack of comparative material (cf. Worthy [20] who found 14 osteological character state differences between these two taxa). Siegel-Causey [18] tentatively retained *L. chalconotus* as a distinct species, sister to a group containing *L. onslowi*, *L. ranfurlyi* and *L. colensoi*. Worthy [10] criticised Siegel-Causey's [18] argument for maintaining separate species status for *L. chalconotus* when *L. carunculatus* was not included in the study.

Based on the regional differentiation detected by Lalas [4] and the absence of apparent differentiation between the bones of *L. chalconotus* and *L. carunculatus*, Worthy [10] suggested that these taxa should be treated as synonyms, with regional morphological variation interpreted as clinal. Worthy [10] showed that the size range of *L. carunculatus* overlapped with *L. chalconotus* from Otago. Worthy [10] further stated that the only difference between the two taxa was the dimorphic plumage in populations of *L. chalconotus*, with the pied morph very similar to *L. carunculatus*, although, he did not consider differences in facial colour. On the basis of these findings and initial genetic conclusions, Schuckard [21] considered it unlikely that *L. chalconotus*, *L. carunculatus* and *L. onslowi* would maintain their specific rank in future taxonomic revisions. However, subsequent genetic analyses of the Phalacrocoracidae by Kennedy and Spencer (unpublished data) indicate strongly that *L. carunculatus* represents a distinct species that is not closely related to either *L. chalconotus* or *L. onslowi*.

In light of distributional, morphological and behavioural data, a detailed molecular reappraisal of *L. chalconotus* systematics seemed overdue. We use both morphological and genetic data to reassess the systematic status of *Leucocarbo* shags from southern New Zealand.

## Materials and Methods

### Ethics statement

Animal ethics permits from the University of Otago's Animal Ethics Committee were not required as samples were only taken from deceased birds. Specimens were not collected from conservation gazetted or private land, and were provided to NJR and MK under Department of Conservation (DoC) conservation management auspices. Specimens are held at the University of Otago's Department of Zoology under a permit to hold material from protected wildlife for genetic analysis (DoC OT-25557-DOA). Permission to sample museum specimens was obtained by NJR and MK from individual institutions (see Table S1 for location and accession details of specimens utilised in this study).

### Source of specimens

Dead, beach-wrecked *L. chalconotus* specimens and additional dead birds (including historical skins) retrieved from breeding colonies or roosting sites across the geographic range of *L. chalconotus* (n = 43, comprising 14 dark-bronze, 18 pied and 11 unrecorded morphotype) were obtained from a variety of sources including DoC and museum collections (Fig. 1; Table S1). *L. onslowi* samples, comprising tissue and Holocene fossil bones (n = 10), were also included to help clarify the status of *L. onslowi* (Fig. 1; Table S1). Samples from sub-Antarctic taxa *L. colensoi* (n = 1) and *L. campbelli* (n = 1) were included as out-groups (Table S1) (see [22]).

### Modern DNA extraction, PCR amplification and DNA sequencing

Whole genomic DNA was extracted from 2 mm^2^ tissue samples following a modified Chelex protocol with 5% Chelex solution, 2 µL Proteinase K (20 mg/mL) and overnight incubation at 56°C [23]. For recent bone samples, DNA was extracted from up to 200 mg of bone powder using the Qiagen DNeasy Tissue Kit following the manufactures instructions with an overnight incubation at 56°C. DNA from recent bone samples was amplified following the methodology for ancient DNA below.

PCR primers were designed to amplify control region one (*CR1*) of the Phalacrocoracidae mitochondrial DNA (mtDNA) genome. In phalacrocoracids and core pelecaniforms there is a duplication of the 3′ end of cytochrome b (*cyt* b) to the 3′ end of the control region (*CR*) [24]–[25]. In *L. chalconotus* there is very low (<20%) sequence similarity between *CR1* and *CR2* compared with other core pelecaniforms (see [25]). *CR1* was amplified using one of several alternate primer pairs: AV16531FND6 (5′ ACCACCARCATHCCCCCYAAATA 3′; Gillian Gibb pers. comm.)/NewCytB R1 (5′ ACAGTTTGATGAAATACCTAGTGGG 3′; this study) (∼1200 bp including primers); AV16531FND6/NewCytB R2 (5′ GATTTTGTCACAGTTTGATGAAATACC 3′; this study) (∼1200 bp including primers); and AV16758FtGlu (5′ TRTGGCYTGAAAARCCRTCGTTG 3′; Gillian Gibb pers. comm.)/NewCytB R2 (∼1000 bp including primers). For one sample (Museum of New Zealand Te Papa Tongarewa (NMNZ) OR.17326) only a partial *CR1* fragment could be amplified using the primer pair AV16531FND6/H10 (5′ GTGAGGTGGACGATCAATAAAT 3′; [26]). Each PCR reaction (10 µL) consisted of: 5 µL of MyFi DNA Polymerase mix (Bioline), 0.5 µL each primer (10 µM), and 1 µL DNA. PCR thermocycling conditions consisted of 94°C 3 min, 40 cycles 94°C 30 s, 50°C 45 s, 72°C 2 min 30 s, followed by a final extension of 72°C 4 min. PCR products were run on a 1% 1× TAE agarose gel. All extractions and PCRs included negative controls. PCR products were purified using an Omega Ultra-Sep PCR clean-up kit, then sequenced bi-directionally using Big Dye Terminator technology and an ABI 3730×l.

### Ancient DNA analysis

All ancient DNA (aDNA) extractions and PCR set up was carried out at the University of Otago in purpose built aDNA (Holocene fossil samples) and historical DNA (historical specimens) laboratories physically isolated from other molecular laboratories [27]. Strict aDNA procedures were followed to minimise contamination of samples with exogenous DNA [28] including the use of negative extraction and PCR controls.

DNA was extracted from up to 250 mg of bone powder sampled from Holocene fossil *L. onslowi* bones following [29]. DNA was extracted from *L. chalconotus* toe pads using the Qiagen DNeasy Blood and Tissue Kit following the manufactures instructions with an overnight incubation at 56°C. Two overlapping fragments of the mtDNA *CR1* were amplified using the primers BESCR1 F1 (5′-GCCACATGATACATTACATG-3′)/R1 (5′-CRCTTATACATAAACTCCTAG -3′) (197 bp including primers) and BESCR1 F2 (5′-CATGTACARACCCATYCCTCCC-3′)/R2 (5′-GTATCCGGTTTCTGAAGTACCAG-3′) (210 bp including primers). Each PCR reaction (20 µL) consisted of: 1 M Betaine (Sigma), 4 mM MgCl_2_ (Life Technologies), 1× Gold Buffer II (Life Technologies), 2.5 mM dNTPs (Bioline), 250 nM each primer, 1.25 U of AmpliTaq Gold DNA Polymerase (Life Technologies), and 2 µL DNA. Unsuccessful PCR's were repeated with 2 U AmpliTaq Gold DNA Polymerase and 4 µL DNA or 2 µL 1∶10 DNA. PCR thermocycling conditions consisted of 94°C 9 min, 60 cycles 94°C 30 s, 50°C 45 s, 72°C 1 min, followed by a final extension of 72°C 10 min. PCR amplification and all downstream procedures were carried out in a modern genetics laboratory.

PCR products were run on a 2% 1× TAE agarose gel. PCR products were purified (using ExoSap (1.5 U ExoI, I U SAP; GE Healthcare) by incubation at 37°C for 30 min and 80°C for 15 min), and sequenced as above, except that bi-directional sequencing was conducted from independent PCR products. When an inconsistency between sequences from an individual was observed due to DNA damage (C-T and G-A transitions), additional PCRs and bi-directional sequencing were conducted, and a majority rule consensus was applied to the independent replicates [30].

### Phylogenetic analysis

Contiguous sequences were constructed using Sequencher (Gencodes) and aligned in MEGA 4.0 [31]. The alignment was trimmed to include 1040 bp of *CR1* sequence data (i.e. excluding the *ND6* and *tRNA Glu* sequence and a short region at the 3′ end of *CR1*). ModelTest was used to determine the most appropriate model of nucleotide substitution under the Akaike Information Criterion. Two datasets were used for phylogenetic analysis: (1) 1040 bp *CR1* (modern samples) and (2) 248 bp *CR1* (modern, recent, historic and ancient) common to all samples (for both of these datasets ModelTest determined the most appropriate model of nucleotide substitution was HKY + I). Bayesian analysis was performed using BEAST v1.7.4 [32] and the Yule birth-death coalescent tree prior. Three independent runs were conducted, consisting of 30 million MCMC generations, sampling tree parameters every 1000 generations, with a burn in of 25%. Phylogenetic convergence was assessed in Tracer, and results analysed in FigTree. DNA sequences are accessioned in GenBank (KJ189963-KJ190017). As well as the Bayesian posterior probabilities (PP), the level of support for the tree topology was evaluated with 1000 bootstrap replicates. Maximum likelihood bootstrap analysis was performed using PhyML [33]. Maximum parsimony (with equal weights and 10 random addition sequence replicates) and neighbour-joining bootstrap analyses were performed using PAUP [34].

### Morphometric analysis

Total length measurements (defined by [35]), of coracoids, humeri, ulnae, carpometacarpi, femora, tibiotarsi and tarsometatarsi were performed using vernier callipers, to the nearest 0.1 mm. The species measured included: *L. colensoi* (n = 15) and *L. chalconotus* from the Otago (n = 32) and Foveaux Strait (n = 8) populations. These bones were sourced from recent and Holocene fossil skeletons housed in New Zealand museum collections (Table S2). Box plots of element length for each species/geographic region were constructed using the program R [36]. Differences in physical size between *L. chalconotus* from Otago and Foveaux Strait, and *L. chalconotus* and *L. onslowi*, were assessed for each measured element individually, using the Mann-Whitney U (MWU) test, implemented in R using the wilcox.test function (Table 1). The MWU test is a non-parametric t-test that compares median values of element length distributions to determine if birds from one population are larger than those from another population. Morphological differentiation was assessed using Principal Component Analysis (PCA) on pooled element lengths (from specimens with no missing data) for *L. chalconotus* from Otago and Foveaux Strait, and *L. onslowi*, using the prcomp function in R.

**Table 1:** Mann-Whitney U test statistics (*U*) and p values (*P*) for assessing size differentiation between *Leucocarbo chalconotus* from Otago and Foveaux Strait, and *L. chalconotus* and *L. onslowi*.

Element	*L. chalconotus* Otago vs Foveaux		*L. chalconotus* vs *L*. *onslowi*	
	*U*	*P*	*U*	*P*
Coracoid	349.50	0.00063	138.00	<10^−9^
Humerus	321.50	0.0033	19.50	<10^−9^
Ulna	278.00	0.0026	24.00	<10^−9^
Carpometacarpus	253.50	0.00037	9.50	<10^−9^
Femur	206.50	0.0081	108.50	<10^−9^
Tibia	300.50	0.00049	59.00	<10^−9^
Tarsometatarsus	272.00	0.00010	39.00	<10^−9^

## Results

DNA sequence alignments for the modern specimens (15 in-group individuals) yielded up to 1040 bp of mtDNA *CR1*. Of the 1040 bp alignment 999 bp were constant, and of the 41 variable sites 27 were parsimony informative. For the 248 bp mtDNA *CR1* fragment (53 in-group individuals) 218 bp were constant, and of the 30 variable sites 25 were parsimony informative. Our phylogenetic analyses of these two datasets strongly supported there being two clades of *Leucocarbo* shag in southern New Zealand (Figs. 2, 3, S1). The first clade comprises dark-bronze and pied morphotypes from Foveaux Strait along with a single dark-bronze-morph beach-wrecked specimen collected from the western shore of Boulder Beach, Otago Peninsula (collected on 22 June 2011 by G. Loh; currently held at the University of Otago's Department of Zoology and will be deposited in the NMNZ collections). The second clade, which in most of the analyses is sister to the Chatham Islands endemic *L. onslowi*, comprises dark-bronze and pied morphotypes sampled from Otago populations. With the exception of maximum likelihood and neighbour joining for the 248 bp dataset, our phylogenetic analyses produced support for the sister group relationship between the *L. chalconotus* Otago lineage and *L. onslowi*. In contrast, maximum likelihood and neighbour joining bootstrapping of the 248 bp dataset produced weak bootstrap support (54% and 58% respectively) for the sister group relationship between the Otago and Foveaux Strait clades (while still supporting the monophyly of each of these groups) (Fig. S2).

For the 248 bp mtDNA *CR1* fragment, the mean percentage divergence between the *L. chalconotus* Foveaux Strait and Otago lineages is 2.6%, whereas the Otago lineage is only 1.8% divergent from *L. onslowi* (compared to 3.5% for the contrast between the Foveaux Strait lineage and *L. onslowi*). The percentage divergence decreases when the longer 1040 bp of mtDNA *CR1* is considered (*L. chalconotus* Foveaux Strait versus Otago 1.2%, Foveaux Strait versus *L. onslowi* 1.8%, Otago versus *L. onslowi* 1.0%). This decrease in mean percentage divergence is because a greater proportion of variable nucleotide positions are contained within the 248 bp *CR1* fragment (∼12% compared with ∼4% in the 1040 bp CR fragment).

**Figure 4:**
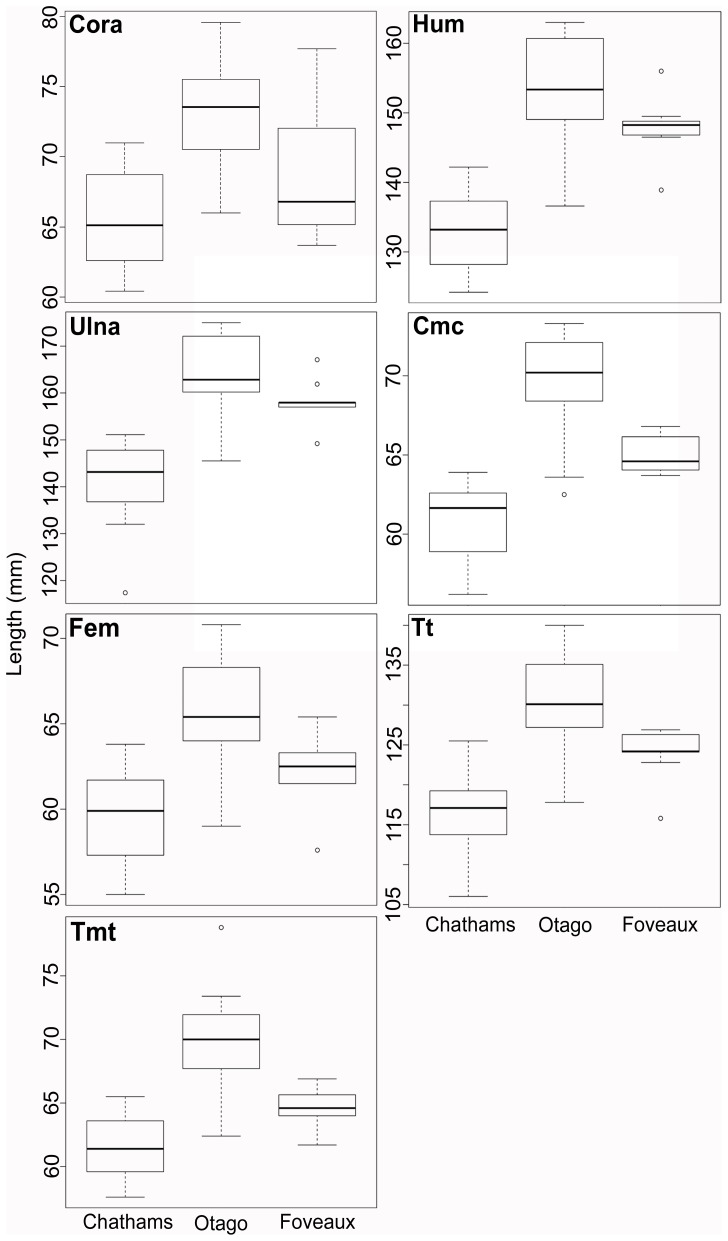
Box plots showing the distribution of *Leucocarbo chalconotus* and *L. onslowi* element lengths (mm). From the median (thick black line) outwards, are the 75th and 25th percentiles (box), the 98th and 2nd percentile (whiskers) and outliers (hollow dots). *L. chalconotus* from Otago are larger on average than Foveaux Strait birds (*P*<0.01; Table 1), and *L. chalconotus* are larger than *L. onslowi* (Table 1).

**Figure 5:**
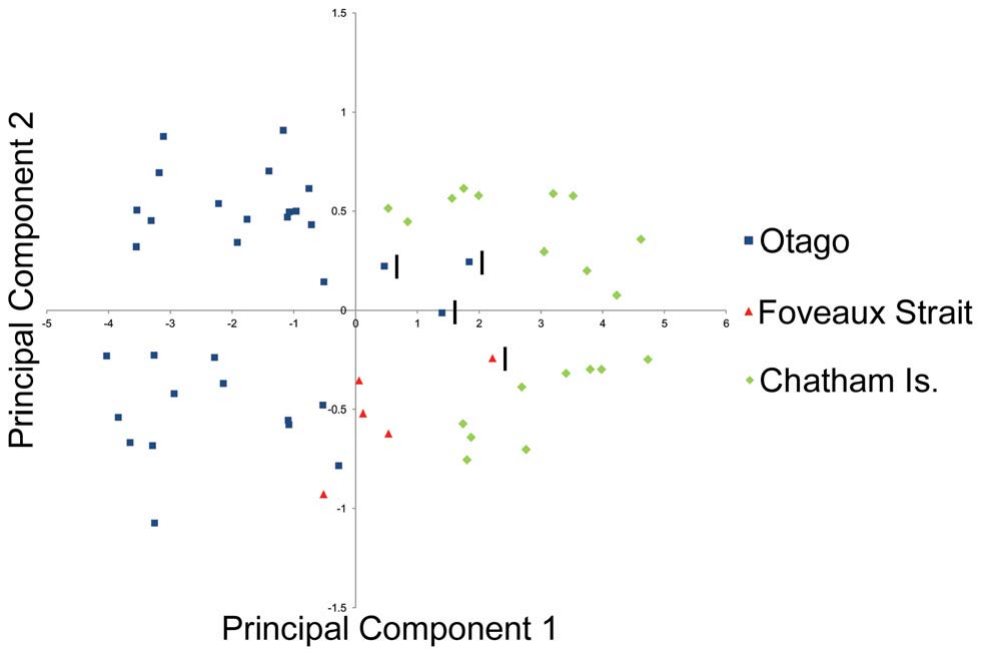
PCA cluster analysis of *Leucocarbo chalconotus* from southern New Zealand, and *L. onslowi*. The analysis was conducted on pooled element lengths (femur, tibiotarsus, tarsometatarsus, coracoid, humerus, ulna, and carpometacarpus). Immature birds are indicated with an ‘I’ on the figure.

Morphometric analysis shows that *L. chalconotus* shags from Otago are larger on average than those from the corresponding Foveaux Strait populations for all elements measured (MWU *P*<0.01; Fig. 4, Table 1). The analysis also showed that, again for all elements, *L. onslowi* is smaller on average than *L. chalconotus* (MWU *P*<0.01; Fig. 4, Table 1). A PCA analysis of pooled element lengths (from specimens with no missing data) revealed substantial morphological differentiation between the three regional population groupings, with just a few immature Otago and Foveaux Strait birds showing morphological overlap with specimens from the Chatham Islands (Fig. 5). Even with these immature birds included, the morphometric differences observed between *L. chalconotus* from Otago and Foveaux Strait, and *L. chalconotus* and *L. onslowi*, are highly significant.

## Discussion

Our phylogenetic analyses show that *Leucocarbo* shags in southern New Zealand comprise two well-supported clades (Otago and Foveaux Strait), each containing both pied and dark-bronze morphs. Surprisingly, however, the combined monophyly of these *L. chalconotus* populations is not well supported, with the Otago lineage typically found to be sister to the Chatham Island endemic, *L. onslowi*, albeit with weak statistical support (a small subset of our analyses do weakly support the monophyly of the two *L. chalconotus* populations). Morphometric analysis indicates that *Leucocarbo* shags from Otago are larger on average than those from Foveaux Strait, corroborating Lalas' [4] observations of substantial size differences between individuals from these regions.

We interpret the specimens from the Southland coast (Oreti Beach and Invercargill) as beach-wrecked individuals from the Foveaux Strait population (Figs. 1–3, S1). Given the prevailing southerly winds in the region, it is entirely plausible that dead individuals from Foveaux Strait will regularly wash up on southern South Island beaches. We also interpret the beach-wrecked specimen from Boulder Beach, Otago Peninsula, as likely of southern origin. This hypothesis is supported by the specimen having scattered papillae, consistent with the Foveaux Strait morphotype [4] (Fig. 1). Juvenile and adult *Leucocarbo* shags are known to travel long distances [6], [13], which, in combination with the direction of the Southland Current, suggests that finding a beach-wreck from the Foveaux Strait population on the southern side of Otago Peninsula is not altogether unexpected. Although we did not sample specimens from every location that the species occur, from external morphology we would also expect extant individuals from Nugget Point, Kinakina Island and The Sisters in the Catlins (plumage proportions reported by [5] are 20–30% pied for these colonies) to represent the Otago lineage (Fig. 1). The specimen from Cooks Head, Milton, just north of Nugget Point, falls within the Otago clade, which is consistent with this suggestion.

Our phylogenetic results, together with consistent differences in relative proportions of plumage morphs and facial carunculation, and concordant differentiation in body size and breeding time [4]–[5], suggest three taxonomic alternatives requiring further investigation that are all consistent with the observed phylogeographic pattern of (Foveaux (Otago, Chatham Islands)) (Figs. 2, 3, S1). Resulting taxonomic classifications depend on differing interpretations of taxonomic philosophy. The first hypothesis is that the Otago and Foveaux Strait lineages of *L. chalconotus* represent phylogenetically and diagnosably distinct taxa (at the species level), rather than the prevailing view that there is a single variable taxon [1]–[2], [10]. This hypothesis also recognises the separate specific status of the rare Chatham Island endemic, *L. onslowi*. *L. onslowi* individuals are morphologically distinct as they are only of the pied morph and smaller on average than *L. chalconotus* (Figs. 4, 5), and their ranges do not overlap [1], [14]. Worthy [20] found 14 osteological character state differences between *L. chalconotus* and *L. onslowi*. *L. onslowi* individuals also differ substantially in facial colouring from *L. chalconotus*. Breeding *L. onslowi* individuals consistently have pronounced bright orange caruncles compared to small bright orange caruncles (Otago) and scattered dark to dull orange papillae (Foveaux Strait and Otago) in *L. chalconotus* [13]. Though more variable, but following a similar pattern to the carunculations, the gular throat patch is bright red in breeding *L. onslowi* individuals, while it ranges from dull to bright orange-red in *L. chalconotus* [13]. Determining whether the Otago and Foveaux Strait lineages of *L. chalconotus* represent diagnosably distinct taxa will require (1) osteological analyses of recent skeletons (identified to morph, age and sex) and Holocene fossil bones to test for the presence of discrete morphological characters; (2) additional morphological analysis of the extent of carunculation to determine whether this can be used as a discrete morphological character; (3) genetic analysis of specimens from un-sampled areas (e.g. Kinakina Island, The Sisters in the Catlins); and (4) ancient DNA analyses of type specimens, Holocene fossil and archaeological material.

The second plausible hypothesis is that *L. chalconotus* may represent a paraphyletic species with strong phylogeographic structure, a consequence of the regional philopatry that is well-documented in this group of birds. Such a finding would not be unexpected, as Joseph and Omland [37], for instance, showed that 44% of Australo-Papuan terrestrial avian taxa that are good biological species are paraphyletic for mtDNA markers. Applying a biological or phylogenetic species concept to taxa with allopatric populations can be problematic, especially when they exhibit site-specific or regional philopatry. Future research will focus on population-genetic analysis of the Otago and Foveaux Strait clades to determine the level of gene flow, population divergence and dynamics between each region [38]–[39]. There are only two single nucleotide polymorphisms (out of over 5000 bp of nuclear DNA) that separate *L. onslowi* and the *L. chalconotus* Otago lineage (Kennedy and Spencer unpublished data). As a consequence, genetic discrimination of the Otago and Foveaux lineages based on nuclear sequences is unlikely.

As a consequence of this second hypothesis, a third hypothesis could be advocated that *L. chalconotus* and *L. onslowi* represent a single, monophyletic taxon with marked phylogeographic structure and possible sub-specific status for the Foveaux, Otago and Chatham Island clades. Nevertheless, this hypothesis is unlikely as *L. onslowi* is morphologically (Figs. 1, 4 and 5) [1], [14], [20] and genetically (Figs. 2, 3, S1) distinct from *L. chalconotus*.

The species-rich radiation of New Zealand blue eyed shags (including *L. ranfurlyi*, *L. campbelli*, *L. colensoi*, *L. onslowi* and *L. carunculatus*), and especially the diversity within *L. chalconotus*, contrasts strongly with the situation for South American/Antarctic blue eyed shags which show less evidence for evolutionary diversification [1]. The phylogeographic partitioning detected within *L. chalconotus* in particular is marked, and such strong structure is rare for Phalacrocoracidae species. Within *Leucocarbo*, Calderon et al. [39] found strong phylogeographic structure and limited gene flow in the Patagonian Rock Shag (*Leucocarbo magellanicus*) using mtDNA *ATPase 6–8* and microsatellites. The Rock Shag contained three mtDNA phylogeographic groups comprising colonies from the Atlantic, Pacific and Fuegian regions of Patagonia. In contrast, there was weaker phylogeographic structure and higher levels of gene flow in the sympatric Imperial Shag (*Leucocarbo atriceps*) [39]. Calderon et al. [39] concluded that Pleistocene glacial cycles, and differences in foraging ecology and non-breeding distribution between the Rock and Imperial Shag contributed to the differing levels of phylogeographic structure. Winney et al. [26] and Marion and Gentil [40] found only moderate phylogeographic structure in the Great Cormorant (*Phalacrocorax carbo*) from Europe using mtDNA *CR1* data, while Barlow et al. [41], using a combination of mtDNA *ND2* and microsatellite data from the European Shag (*Phalacrocorax aristotelis*), found only weak population-genetic structure, despite high levels of philopatry, rare dispersal events and recognised subspecies. Waits et al. [42], using mtDNA *12S rRNA*, *16S rRNA*, *COIII-ND3*, *cyt* b and *CR*, found no phylogeographic structure or restricted gene flow among populations or between subspecies of the Double-crested Cormorant (*Phalacrocorax auritus*) in eastern North America. However, Mercer et al. [43] used *CR* data and found there was phylogeographic structure between the Alaskan Double-crested Cormorant and those from eastern North America. In another coastal bird taxon, the Dusky Seaside Sparrow (*Ammodramus maritimus*), Avise and Nelson [44] detected substantial phylogeographic structuring of populations from eastern and southern North America, delineated by the Florida peninsula. It should be noted that the genetic data used in our study are from rapidly evolving mtDNA *CR1*, rather than the more slowly evolving ‘barcoding’ COI marker often used in bird systematics (e.g. [45] cf. [46]). However, there are several diagnosably distinct avian taxa than cannot be distinguished using slowly evolving mtDNA markers but can be identified using the faster evolving *CR* (e.g. *cyt* b versus *CR* in *Cyanoramphus* parakeets [47]–[48]).

## Conclusions

In this study we have shown that the Stewart Island Shag (*L. chalconotus*) groups into two geographically separate (Otago and Foveaux Strait) reciprocally monophyletic clades. In the majority of our analyses the Otago clade groups with the Chatham Island Shag (*L. onslowi*), although there is some support for the Otago and Foveaux Strait clades grouping together. Further data are required to resolve, with greater certainty, the relationship between the Otago, Foveaux Strait, and Chatham Island groups. Irrespective of the relationships between these groups inferred from our phylogenies, arguments could be made for different taxonomic arrangements of these clades, namely (1) splitting *L. chalconotus* into two units (with *L. onslowi* as a third); (2) leaving *L. chalconotus* as a single unit (with *L. onslowi* as a second); and (3) subsuming *L. onslowi* within *L. chalconotus*. We do not support this last option, however, given the osteological and morphological uniqueness of *L. onslowi* [13], [19]. Further studies are required to evaluate which of these options best represents the level of taxonomic distinctiveness of these clades.

## Supporting Information

Figure S1 **Neighbour joining phylogeny of** ***Leucocarbo chalconotus*** **and** ***L. onslowi*** **shags based on 1040 bp mtDNA** ***CR1.***
(PDF)

Figure S2 **Neighbour joining phylogeny of** ***Leucocarbo chalconotus*** **and** ***L. onslowi*** **shags based on 248 bp mtDNA** ***CR1.***
(PDF)

Table S1 ***Leucocarbo*** **shag specimens used for genetic analysis.**
(PDF)

Table S2 **Element maximum length measurements (to the nearest 0.1 mm) of Chatham Island Shag (** ***Leucocarbo onslowi*** **) and Stewart Island Shag (** ***L. chalconotus*** **) from Otago and Foveaux Strait populations.**
(PDF)

## References

[1] Marchant S, Higgins PJ (1990) Handbook of Australian, New Zealand and Antarctic birds (Volume 1, Part B Pelican to Ducks). Melbourne: Oxford University Press. 1400 p.

[2] Gill BJ, Bell BD, Chambers GK, Medway DG, Palma RL, et al.. (2010) Checklist of the birds in New Zealand, Norfolk and Macquarie Islands, and the Ross Dependency Antarctica (4th edition). Wellington: Te Papa Press in association with the Ornithological Society of New Zealand. 512 p.

[3] Blackburn A (1968) The birdlife of Codfish Island. Notornis 15: 51–65.

[4] Lalas C (1983) Comparative feeding ecology of New Zealand marine shags (Phalacrocoracidae). PhD thesis, University of Otago, Zoology Department. 291 p.

[5] Lalas C, Perriman L (2009) Nest counts of Stewart Island shags/mapua (*Leucocarbo chalconotus*) in Otago. DoC Research and Development Series 314: 1–30.

[6] Crossland AC (2012) A review of the current range of Stewart Island Shag (*Leucocarbo chalconotus*) and two records from Lake Ellesmere, Canterbury. Notornis 59: 71–73.

[7] Buller WL (1888) A history of the birds of New Zealand (2nd edition, Volume II). London: The author.

[8] Watt JPC (1973) Notes on Whero Island and other roosting and breeding stations of the Stewart Island shag (*Leucocarbo carunculatus chalconotus*). Notornis 22: 265–272.

[9] Falla RA, Sibson RB, Turbott EG (1978) The new guide to the birds of New Zealand and outlying islands. Auckland: Collins. 247 p.

[10] Worthy TH (1996) Holocene populations of shags *Leucocarbo* spp. in the far north, New Zealand. N. Z. J. Zool. 23: 89–95.

[11] Worthy TH (1998) A remarkable fossil and archaeological avifauna from Marfells Beach, Lake Grassmere, South Island, New Zealand. Rec. Cant. Mus. 12: 79–176.

[12] Wilmshurst JM, Anderson AJ, Higham TFG, Worthy TH (2008) Dating the late prehistoric dispersal of Polynesians to New Zealand using the commensal Pacific rat. Proc. Natl. Acad. Sci. U. S. A. 105: 7676–7680. 10.1073/pnas.0801507105 PMC2409139 18523023

[13] Heather BD, Robertson HA (2005) The fieldguide to the birds of New Zealand. Auckland: Viking. 440 p.

[14] Scofield RP, Stephenson B (2013) A photographic guide to the birds of New Zealand. Auckland: Auckland University Press. 552 p.

[15] Peters JL (1931) Check-list of birds of the world (Volume 1). Cambridge: Harvard University Press.

[16] Kinsky FC (1970) Annotated checklist of the birds of New Zealand (2nd edition). Wellington: Ornithological Society of New Zealand Incorporated. 96 p.

[17] Falla RA (1932) New Zealand cormorants in the collection of the Auckland Museum, with notes on field observations. Rec. Auck. Inst. Mus. 1: 139–154.

[18] Siegel-Causey D (1988) Phylogeny of the Phalacrocoracidae. Condor 90: 885–905.

[19] Turbott EG (1990) Checklist of the birds of New Zealand and the Ross Dependency, Antarctica. Auckland: Ornithological Society of New Zealand Random Century New Zealand. 247 p.

[20] Worthy TH (2011) Descriptions and phylogenetic relationships of a new genus and two species of Oligo-Miocene cormorants (Aves: Phalacrocoracidae) from Australia. Zool. J. Linn. Soc. 163: 277–314.

[21] Schuckard R (2006) Population status of the New Zealand king shag (*Leucocarbo carunculatus*). Notornis 53: 297–307.

[22] Holland BR, Spencer HG, Worthy TH, Kennedy M (2010) Identifying cliques of convergent characters: Concerted evolution in the cormorants and shags. Syst. Biol. 59: 433–445. 10.1093/sysbio/syq023 20547779

[23] Walsh PS, Metzger DA, Higuchi R (2013) Chelex 100 as a medium for simple extraction of DNA for PCR-based typing from forensic material. Biotechniques 54: 134–139. 1867860

[24] Morris-Pocock JA, Taylor SA, Brit TP, Friesen VL (2010) Concerted evolution of duplicated control regions in three related seabird species. BMC Evol. Biol. 10: 14. 10.1186/1471-2148-10-14 PMC2820450 20074358

[25] Gibb GC, Kennedy M, Penny D (2013) Beyond phylogeny: Pelecaniform and ciconiiform birds, and long-term niche stability. Mol. Phylogenet. Evol. 68: 229–238. 10.1016/j.ympev.2013.03.021 23562800

[26] Winney BJ, Litton CD, Parkin DT, Feare CJ (2001) The subspecific origin of the inland breeding colonies of the cormorant Phalacrocorax carbo in Britain. Heredity 86: 45–53. 11298814 10.1046/j.1365-2540.2001.00807.x

[27] Knapp M, Clarke AC, Horsburgh KA, Matisoo-Smith EA (2012) Setting the stage – building and working in an ancient DNA laboratory. Ann. Anat. 194: 3–6. 10.1016/j.aanat.2011.03.008 21514120

[28] Cooper A, Poinar HN (2000) Ancient DNA: Do it right or not at all. Science 289: 1139. 10970224 10.1126/science.289.5482.1139b

[29] Rohland N, Siedel H, Hofreiter M (2010) A rapid column-based ancient DNA extraction method for increased sample throughput. Mol. Ecol. Resour. 10: 677–683. 10.1111/j.1755-0998.2009.02824.x 21565072

[30] Brotherton P, Endicott P, Sanchez JJ, Beaumont M, Barnett R, et al (2007) Novel high-resolution characterization of ancient DNA reveals C > U-type base modification events as the sole cause of *post mortem* miscoding lesions. Nucleic Acids Res. 35: 5717–5728. 10.1093/nar/gkm588 PMC2034480 17715147

[31] Kumar S, Tamura K, Nei M (2004) MEGA3: Integrated software for molecular evolutionary genetics analysis and sequence alignment. Brief. Bioinformatics 5: 150–163. 10.1093/bib/5.2.150 15260895

[32] Drummond AJ, Rambaut A (2007) BEAST: Bayesian evolutionary analysis by sampling trees. BMC Evol. Biol. 7: 214. 10.1186/1471-2148-7-214 PMC2247476 17996036

[33] Guindon S, Dufayard JF, Lefort V, Anisimova M, Hordijk W, et al (2010) New algorithms and methods to estimate maximum-likelihood phylogenies: Assessing the performance of PhyML 3.0. Syst. Biol. 59: 307–321. 10.1093/sysbio/syq010 20525638

[34] Swofford DL (2002) PAUP*. Phylogenetic Analysis Using Parsimony (*and Other Methods). Sunderland: Sinauer Associates.

[35] Driesch A (1976) A guide to the measurements of animal bones from archaeological sites. Peabody Museum Bulletin 1.

[36] R Development Core Team (2008) R: A language and environment for statistical computing. Vienna: R Foundation for Statistical Computing.

[37] Joseph L, Omland KE (2009) Phylogeography: Its development and impact in Australo-Papuan ornithology with special reference to paraphylly in Australian birds. Emu 109: 1–23.

[38] Peterson BK, Weber JN, Kay EH, Fisher HS, Hoekstra HE (2012) Double digest RADseq: An inexpensive method for de novo SNP discovery and genotyping in model and non-model species. PLoS One 7: e37135. 22675423 10.1371/journal.pone.0037135 PMC3365034

[39] Calderon L, Quintana F, Cabanne GS, Lougheed SC, Tubaro PL (2014) Phylogeography and genetic structure of two Patagonian shag species (Aves: Phalacrocoracidae). Mol. Phylogenet. Evol. 72: 42–53. 10.1016/j.ympev.2013.12.011 24418531

[40] Marion L, Gentil J (2006) Ecological segregation and population structuring of the Cormorant *Phalacrocorax carbo* in Europe, in relation to the recent introgression of continental and marine subspecies. Evol. Ecol. 20: 193–216.

[41] Barlow EJ, Daunt F, Wanless S, Alvarez D, Reid JM, et al (2011) Weak large-scale population genetic structure in a philopatric seabird, the European Shag *Phalacrocorax aristotelis* . Ibis 153: 768–778.

[42] Waits JL, Avery ML, Tobin ME, Leberg PL (2003) Low mitochondrial DNA variation in Double-crested Cormorants in eastern North America. Waterbirds 26: 196–200.

[43] Mercer DM, Haig SM, Roby DD (2013) Phylogeography and population genetic structure of double-crested cormorants (*Phalacrocorax auritus*). Conserv. Genet. 14: 823–836.

[44] Avise JC, Nelson WS (1988) Molecular genetic relationships of the extinct Dusky Seaside Sparrow. Science 243: 646–648. 10.1126/science.243.4891.646 17834232

[45] Lambert DM, Baker A, Huynen L, Haddrath O, Hebert PDN, et al (2005) Is a large-scale DNA-based inventory of ancient life possible? J. Hered. 96: 279–284. 10.1093/jhered/esi035 15731217

[46] Bunce M, Worthy TH, Phillips MJ, Holdaway RN, Willerslev E, et al (2009) The evolutionary history of the extinct ratite moa and New Zealand Neogene palaeogeography. Proc. Natl. Acad. Sci. U. S. A. 106: 20646–20651. 10.1073/pnas.0906660106 PMC2791642 19923428

[47] Boon WM, Kearvell JC, Daugherty CH, Chambers GK (2000) Molecular systematics of New Zealand *Cyanoramphus* parakeets: conservation of orange-fronted and Forbes' parakeets. Bird Conserv. Int. 10: 211–239.

[48] Boon WM, Robinet O, Rawlence N, Bretagnolle V, Norman JA, et al (2008) Morphological, behavioural and genetic differentiation within the Horned Parakeet (*Eunymphicus cornutus*) and its affinities to *Cyanoramphus* and *Prosopeia* . Emu 108: 251–260.

