# Supplementary material for: Strong Phylogeographic Structure in a Sedentary Seabird, the Stewart Island Shag (Leucocarbo chalconotus)
Source: PLoS One. 2014 Mar 10;9(3):e90769. doi: 10.1371/journal.pone.0090769 (PMC3948693; doi:10.1371/journal.pone.0090769)
Supplement: Figure S1 — Neighbour joining phylogeny of Leucocarbo chalconotus and L. onslowi shags based on 1040 bp mtDNA CR1. (PDF) [file pone.0090769.s001.pdf]

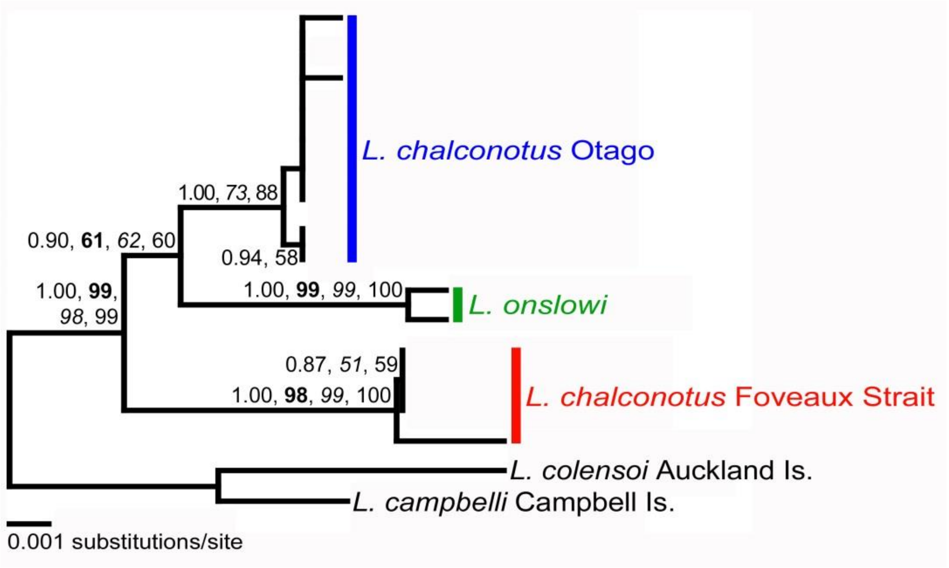

Figure S1. Neighbour joining phylogeny of *Leucocarbo chalconotus* and *L. onslowi* based on 1040 bp mtDNA *CR1*. Branch lengths are proportional to the number of substitutions. The phylogeny was generated in PAUP using the HKY + I model of nucleotide substitution with 1000 bootstrap replicates. The phylogeny was rooted using the Auckland Island Shag (*L. colensoi*) and Campbell Island Shag (*L. campbelli*). For clarity, only bootstrap support (**bold**: maximum likelihood; *italics*: maximum parsimony; Roman: neighbour joining) and posterior probability values for major clades are shown (> 50% bootstrap and > 0.60 posterior probability support).
