# Supplementary material for: Strong Phylogeographic Structure in a Sedentary Seabird, the Stewart Island Shag (Leucocarbo chalconotus)
Source: PLoS One. 2014 Mar 10;9(3):e90769. doi: 10.1371/journal.pone.0090769 (PMC3948693; doi:10.1371/journal.pone.0090769)
Supplement: Table S1 — Leucocarbo shag specimens used for genetic analysis. (PDF) [file pone.0090769.s003.pdf]

Table S1 *Leucocarbo* shag specimens used for genetic analysis.

Species	Collection location	Collection date	Plumage morph	Voucher information	GenBank no.
Leucocarbo chalconotus Otago	Oamaru	Modern tissue	Bronze	OZD SIS B1	KJ189977
L. chalconotus Otago	Karitane	Modern tissue	Pied	OZD SIS K1	KJ189978
L. chalconotus Otago	Warrington	Modern tissue	Pied	OZD SIS O1	KJ189979
L. chalconotus Otago	Long Beach	Modern tissue	Pied	OZD SIS L5	KJ189982
L. chalconotus Otago	Aramoana	Modern tissue	Bronze	OZD SIS L4	KJ189976
L. chalconotus Otago	Sandfly Bay	Modern tissue	Bronze	OM Av8958	KJ189984
L. chalconotus Otago	Taiaroa Head	Modern tissue	Pied	NMNZ OR.17326	KJ189985
L. chalconotus Otago	Taiaroa Head	Recent skeleton (1968)	-	OAD FA141	KJ189987
L. chalconotus Otago	Taiaroa Head	Recent skeleton (1969)	-	OAD FA142	KJ189988
L. chalconotus Otago	Papanui Beach	Recent skeleton (1978)	Pied	OAD FC109	KJ189986
L. chalconotus Otago	Allans Beach	Modern tissue	-	OZD SIS 11	KJ189975
L. chalconotus Otago	Wharekakahu Is.	Modern tissue	Bronze	OZD SIS L1	KJ189980
L. chalconotus Otago	Wharekakahu Is.	Modern tissue	Pied	OZD SIS L2	KJ189981
L. chalconotus Otago	Cooks Head, Milton	Modern tissue	Pied	AM Tax04-85	KJ189983
L. chalconotus Foveaux Strait	Boulder Beach	Modern tissue	Bronze	OZD SIS L6	KJ189989
L. chalconotus Foveaux Strait	Invercargill	Historic skin (1984)	Pied	NMNZ OR.8925	KJ189995
L. chalconotus Foveaux Strait	Oreti Beach	Modern tissue	-	OZD SIS E1	KJ189991
L. chalconotus Foveaux Strait	Paterson Inlet	Modern tissue	-	OZD SIS C1	KJ189990
L. chalconotus Foveaux Strait	Paterson Inlet	Modern tissue	-	OZD SIS C2	KJ189992
L. chalconotus Foveaux Strait	Paterson Inlet	Historic skin (1979)	Pied	NMNZ OR.21469	KJ190009
L. chalconotus Foveaux Strait	Foveaux Strait	Recent skeleton	Pied	AM LB10371	KJ190006
L. chalconotus Foveaux Strait	Foveaux Strait	Recent skeleton	-	AM LB11215	KJ190007
L. chalconotus Foveaux Strait	Seal Rocks, Ruapuke Is.	Historic skin (1930)	Pied	CM Av609	KJ189994
L. chalconotus Foveaux Strait	Seal Rocks, Ruapuke Is.	Historic skin (1930)	Bronze	NMNZ OR.16684	KJ190000
L. chalconotus Foveaux Strait	Seal Rocks, Ruapuke Is.	Historic skin (1930)	Pied	NMNZ OR.16683	KJ190001
L. chalconotus Foveaux Strait	Seal Rocks, Ruapuke Is.	Historic skin (1930)	Pied	CM Av2234	KJ190012
L. chalconotus Foveaux Strait	Seal Rocks, Ruapuke Is.	Historic skin (1930)	Bronze	CM Av612	KJ190015
L. chalconotus Foveaux Strait	Seal Rocks, Ruapuke Is.	Historic skin (1930)	Bronze	CM Av615	KJ190016
L. chalconotus Foveaux Strait	Ruapuke Is.	Historic skin	Bronze	CM Av1480	KJ190013
L. chalconotus Foveaux Strait	Ruapuke Is.	Historic skin (1930)	Pied	CM Av610	KJ190014
L. chalconotus Foveaux Strait	Kanetetoe Is.	Historic skin (1932)	Pied	CM Av607	KJ189993
L. chalconotus Foveaux Strait	Kanetetoe Is.	Historic skin (1932)	Bronze	CM Av614	KJ190011
L. chalconotus Foveaux Strait	Kanetetoe Is.	Historic skin (1932)	Bronze	CM Av828	KJ190017
L. chalconotus Foveaux Strait	Northeast Stewart Is.	Historic skin (1983)	Pied	NMNZ OR.22973	KJ190010
L. chalconotus Foveaux Strait	Port Pegasus	Historic skin (1972)	Pied	NMNZ OR.16640	KJ189997
L. chalconotus Foveaux Strait	Port Pegasus	Historic skin (1972)	Bronze	NMNZ OR.17109	KJ190004
L. chalconotus Foveaux Strait	Port Pegasus	Historic skin (1972)	Bronze	NMNZ OR.17018	KJ190008
L. chalconotus Foveaux Strait	Broad Passage, Port Pegasus	Historic skin (1972)	Bronze	NMNZ OR.17017	KJ190005
L. chalconotus Foveaux Strait	Easy Harbour	Historic skin (1972)	Pied	NMNZ OR.17019	KJ190003
L. chalconotus Foveaux Strait	Shag Rock, Easy Harbour	Historic skin (1972)	Bronze	NMNZ OR.17108	KJ189996
L. chalconotus Foveaux Strait	Stewart Is.	Historic skin (1891)	Pied	CM Av1439	KJ189998
L. chalconotus Foveaux Strait	Stewart Is.	Historic skin (1896)	Bronze	CM Av1478	KJ189999
L. chalconotus Foveaux Strait	Stewart Is.	Historic skin (1929)	Pied	NMNZ OR.14120	KJ190002

Leucocarbo onslowi	Chatham Is.	Modern tissue	Pied	OZD CIS B2	KJ189965
L. onslowi	Chatham Is.	Modern tissue	Pied	OZD CIS B1	KJ189966
L. onslowi	Chatham Is.	Recent skeleton	Pied	OAD FA978	KJ189967
L. onslowi	Chatham Is.	Holocene fossil	-	NMNZ S.24089.1	KJ189968
L. onslowi	Chatham Is.	Holocene fossil	-	NMNZ S.28843.1	KJ189970
L. onslowi	Chatham Is.	Holocene fossil	-	NMNZ S.29457.1	KJ189971
L. onslowi	Chatham Is.	Holocene fossil	-	NMNZ S.28311.1	KJ189974
L. onslowi	East of Ngatikitiki, Chatham Is.	Holocene fossil	-	NMNZ S.31923.1	KJ189969
L. onslowi	Motutapu Pt, Pitt Is.	Holocene fossil	-	NMNZ S.45634.1	KJ189972
L. onslowi	Kaingaroa, Chatham Is.	Holocene fossil	-	NMNZ S.31714.1	KJ189973
Leucocarbo colensoi	Auckland Is.	Modern tissue	Pied	OZD AIS1	KJ189963
Leucocarbo campbelli	Campbell Is.	Modern tissue	Pied	OZD CIS1	KJ189964

Abbreviations are as follows: AM: Auckland Museum, Auckland; CM: Canterbury Museum, Christchurch; NMNZ: Museum of New Zealand Te Papa Tongarewa, Wellington; OM: Otago Museum, Dunedin; OAD: University of Otago Department of Anthropology and Archaeology, Dunedin; OZD: University of Otago Zoology Department, Dunedin.
