# Supplementary material for: Strong Phylogeographic Structure in a Sedentary Seabird, the Stewart Island Shag (Leucocarbo chalconotus)
Source: PLoS One. 2014 Mar 10;9(3):e90769. doi: 10.1371/journal.pone.0090769 (PMC3948693; doi:10.1371/journal.pone.0090769)
Supplement: Table S2 — Element maximum length measurements (to the nearest 0.1 mm) of Chatham Island Shag ( Leucocarbo onslowi ) and Stewart Island Shag ( L. chalconotus ) from Otago and Foveaux Strait populations. (PDF) [file pone.0090769.s004.pdf]

Table S2 Element maximum length measurements (to the nearest 0.1 mm) of Chatham Island Shag (*Leucocarbo onslowi*) and Stewart Island Shag (*L. chalconotus*) from Otago and Foveaux Strait populations.

Voucher Information	Collection Location	Clade	Fem	Tib	Tmt	Hum	Ulna	Cmc	Cora
CM Av19499	Otari Beach	Foveaux	65.4	126.9	66.9	149.5	161.9	66.8	65.3
NMNZ OR.36001	Stewart Is.	Foveaux	61.5	125.1	64.2	148.2	158	64.0	65.0
CM Av32413	Stewart Is.	Foveaux	57.6	115.8	61.7	138.9	149.2	63.7	63.7
CM Av32724	Stewart Is.	Foveaux	63.3	122.8	65.2	148.8	158.0	66.5	67.1
CM Av26066	Stewart Is.	Foveaux	62.9	126.3	66.1	148.3	157.1	64.1	66.5
AM LB10371	Foveaux Strait	Foveaux	60.7	119.9	64.6	140.2	153.6	65.4	69.8
AM LB11215	Foveaux Strait	Foveaux	64.5	125.5	66.8	148.0	158.4	68.7	74.3
OAD PELS-59	Chalky Is.	Foveaux	67.9	136.0	72.7	160.8	174.6	73.3	77.7
NMNZ OR.26221	Oamaru	Otago	70.2	137.7	72.7	161.5	174.0	73.2	72.6
CM Av36705	Oamaru	Otago	68.1	133.5	70.9	159.0	173.0	72.5	72.4
NMNZ OR.29029	Hampden Beach	Otago	65.2	124.9	66.0	147.8	158.8	67.5	73.6
NMNZ OR.29015	Hamden Beach	Otago	64.9	128.0	66.3	149.1	161.5	68.6	73.5
NMNZ OR.27785	Hampden Beach	Otago	66.4	130.5	68.5	152.0	163.0	68.5	74.9
NMNZ OR.29559	Hampden Beach	Otago	64.2	128.3	67.5	147.3	160.0	67.4	73.7
NMNZ OR.29036	Hampden Beach	Otago	61.8	128.3	68.1	146.7	157.5	67.0	71.3
NMNZ OR.27780	Hampden Beach	Otago	64.5	127.6	67.0	151.5	162.7	69.5	75.3
NMNZ OR.27779	Hampden Beach	Otago	64.4	124.6	68.2	149.0	162.6	68.3	73.8
NMNZ OR.29014	Moeraki Boulders	Otago	70.0	134.0	72.0	161.4	172.7	71.7	77.5
NMNZ OR.29013	Moeraki Boulders	Otago	64.3	126.0	67.1	149.1	160.2	69.1	76.0

OM Av884	Otago Peninsula	Otago	64.7	129.1	68.3	151.0	162.0	69.0	68.3
OM Av885	Otago Peninsula	Otago	65.4	133.0	70.5	157.0	167.0	71.2	72.4
OM Av1843	Otago Peninsula	Otago	68.3	135.9	72.4	162.0	172.0	73.3	71.9
OM Av1857	Otago Peninsula	Otago	68.9	136.1	71.9	163.0	175.0	72.7	69.7
OM Av1844	Otago Peninsula	Otago	67.6	135.1	72.1	160.0	174.0	72.8	74.2
NMNZ 611-S	Otago Peninsula	Otago	64.1	126.6	69.4	151.6	160.2	70.6	68.5
NMNZ OR.23706	Otago Peninsula	Otago	61.8	126.4	68.7	147.2	158.9	68.0	66.0
NMNZ OR.15275	Otago Peninsula	Otago	62.6	125.4	67.9	148.9	160.9	68.8	67.8
NMNZ OR.25845	Otago Peninsula	Otago	70.5	140.0	73.4	162.0	173.0	73.3	75.7
NMNZ OR.26357	Otago Peninsula	Otago	70.8	138.5	72.6	163.0	175.0	72.7	73.6
OAD FA142	Otago Peninsula	Otago	68.0	132.8	71.0	157.3	170.2	71.2	79.6
OAD AN992	Otago Peninsula	Otago	69.0	134.9	70.3	160.7	171.6	71.6	77.6
OAD FC510	Otago Peninsula	Otago	59.0	117.8	62.4	136.6	148.5	63.6	67.0
OAD FC109	Otago Peninsula	Otago	66.5	129.7	69.8	155.4	167	70.0	76.1
OAD FB693	Otago Peninsula	Otago	61.7	120.3	64.8	144.4	156.2	65.8	69.4
OAD FB223	Otago Peninsula	Otago	64.0	128.8	67.0	148.7	158.4	65.2	73.0
OAD FC114	Otago Peninsula	Otago	61.5	119.2	65.5	139.1	145.5	62.5	66.9
OAD PELS25	Otago Peninsula	Otago	67.4	137.4	72.6	162.4	172.0	70.7	78.8
OAD FA332	Otago Peninsula	Otago	69.2	132.7	70.0	160.7	172.4	70.2	78.6
OAD FB694	Otago Peninsula	Otago	63.3	129.1	78.8	156.0	160.9	68.2	72.9
OAD AN993	Otago Peninsula	Otago	66.1	128.2	70.0	152.0	163.1	71.0	74.6
CM Av12753	Chatham Is.	Chatham	61.9	119.9	63.5	137.6	146.6	62.6	62.0
CM Av6890	Chatham Is.	Chatham	59.9	115.1	61.8	133.2	144.5	61.3	60.4

CM Av13841	Chatham Is.	Chatham	62.2	118.7	63.9	138.0	146.7	62.3	63.1
CM Av27927	Chatham Is.	Chatham	62.8	118.9	64.3	135.7	144.4	61.7	62.6
NMNZ OR.24430	Chatham Is.	Chatham	55.0	109.2	58.3	124.2	134.0	56.5	62.6
NMNZ OR.21496	Chatham Is.	Chatham	60.0	119.6	62.9	137.3	147.8	62.5	69.3
NMNZ OR.15496	Chatham Is.	Chatham	59.2	117.2	61.5	136.3	145.9	62.7	68.4
NMNZ OR.15498	Chatham Is.	Chatham	60.2	118.3	61.6	136.6	148.0	62.4	69.0
NMNZ OR.27191	Chatham Is.	Chatham	63.0	124.1	64.5	142.2	151.1	63.1	71.0
NMNZ OR.26358	Chatham Is.	Chatham	57.7	114.0	60.4	129.5	138.1	59.6	66.4
NMNZ OR.26435	Chatham Is.	Chatham	58.0	110.3	59.0	130.5	138.0	59.1	65.5
NMNZ S.28311	Chatham Is.	Chatham	56.5	110.0	58.6	126.3	134.6	58.6	61.7
OAD FA979	Chatham Is.	Chatham	58.6	114.7	60.5	131.3	138.1	60.2	65.1
OAD FA977	Chatham Is.	Chatham	56.7	115.0	57.6	127.9	139.4	58.8	63.1
AM LB013673	Pitt Is.	Chatham	62.0	122.0	64.2	139.9	150.4	63.9	70.0

Specimens were sourced from recent and Holocene fossil skeletons housed in New Zealand museum collections. Abbreviations are as follows: Fem: Femur; Tib: Tibiotarsus; Tmt: Tarsometatarsus; Hum: Humerus; Ulna: Ulna; Cmc: Carpometacarpus; Cora: Coracoid; AM: Auckland Museum, Auckland; CM: Canterbury Museum, Christchurch; NMNZ: Museum of New Zealand Te Papa Tongarewa, Wellington; OM: Otago Museum, Dunedin; OAD: University of Otago Department of Anthropology and Archaeology, Dunedin.
